# Breast cancer in Tanzanian, black American, and white American women: An assessment of prognostic and predictive features, including tumor infiltrating lymphocytes

**DOI:** 10.1371/journal.pone.0224760

**Published:** 2019-11-08

**Authors:** Alex Mremi, Gloria Broadwater, Kahima Jackson, Patrick Amsi, Cosmas Mbulwa, Terry Hyslop, Cecilia Ong, Allison Hall

**Affiliations:** 1 Department of Pathology, Kilimanjaro Christian Medical University College, Moshi, Tanzania; 2 Department of Biostatistics and Bioinformatics, Duke University Medical Center, Durham, North Carolina, United States of America; 3 Department of Pathology, Bugando Medical Centre, Mwanza, Tanzania; 4 Department of Surgery, Duke University Medical Center, Durham, North Carolina, United States of America; 5 Department of Pathology, Duke University Medical Center, Durham, North Carolina, United States of America; Qatar University College of Medicine, QATAR

## Abstract

**Introduction:**

Breast cancer is a major cause of morbidity and mortality for women in Sub-Saharan Africa and for black American women. There is evidence that the pathologic characteristics of breast cancers in both African women and black American women may differ from their counterparts of European ancestry. However, despite the great burden of disease, data on pathologic features of breast carcinoma in Sub-Saharan Africa is limited and often contradictory. This lack of information makes it difficult to prioritize resource use in efforts to improve breast cancer outcomes in the region.

**Methods:**

We examined consecutive cases of breast cancer in Tanzanian women (n = 83), black American women (n = 120), and white American women (n = 120). Each case was assessed for tumor type, grade, mitotic count, ER and HER2 status, and tumor infiltrating lymphocyte involvement.

**Results:**

The Tanzanian subjects were younger and had higher stage tumors than the subjects in either American group. Breast cancers in the Tanzanian and black American groups were more likely to be high grade (p = 0.008), to have a high mitotic rate (p<0.0001), and to be ER-negative (p<0.001) than the tumors in the white American group. Higher levels of tumor infiltrating lymphocyte involvement were seen among Tanzanian and black American subjects compared to white American subjects (p = 0.0001). Among all subjects, tumor infiltrating lymphocyte levels were higher in tumors with a high mitotic rate. Among Tanzanian and black American subjects, tumor infiltrating lymphocyte levels were higher in ER-negative tumors. These findings have implications for treatment priorities for breast cancer in Tanzania and other Sub-Saharan African countries.

## Introduction

In Sub-Saharan Africa, breast cancer is currently the most common cancer in women and the second leading cause of cancer mortality [1]. Breast cancer represents one quarter of all cancers in women in the region and causes one fifth of all cancer deaths, with approximately 115,000 new cases and 54,000 deaths per year. Although the incidence of breast cancer in the region is lower than in higher income countries, it is rising rapidly as life expectancy increases and as the population adopts a more western lifestyle in terms of diet, exercise and childbearing patterns. Recent studies of cancer registries in Zimbabwe and Uganda showed an annual increase in breast cancer incidence of 4–5% [2, 3].

Similarly, breast cancer is also the most common cancer and the third most common cause of cancer mortality in black American women [4]. In the past, the incidence of breast cancer in black American women was lower than in white American women. However, while the incidence of breast cancer in white American women has been stable in recent years, the incidence of breast cancer in black American women has been rising, such that the incidence is now essentially the same in both groups [5].

The prognosis for a woman diagnosed with breast cancer in Sub-Saharan Africa is much poorer than for her counterparts in higher resource countries. Data from tumor registries in the Gambia and Uganda disclose 5 year survival rates of only 12% and 46%, respectively [6]. In the United States, overall 5 year survival for breast cancer is 90% [4]. However, breast cancer mortality rates for black American women are 39% higher than for white American women [7].

There are multiple causes of these disparities. In Sub-Saharan Africa, a significant part of the survival discrepancy is related to diagnosis at a more advanced stage, due to lack of breast cancer awareness and the absence of screening programs, and limited access to the multimodality treatments that are available in higher resource environments [8]. Within the United States, socioeconomic disadvantages also have a negative impact on breast cancer outcomes for black American women, resulting in a later stage at diagnosis and lower rates of comprehensive multidisciplinary care, relative to white American women [9–11].

There is also evidence of differences in intrinsic tumor characteristics among groups with African versus European ancestry. Specifically, multiple studies have shown that breast cancers in women in Sub-Saharan Africa arise at a younger age and are higher grade than breast cancers in women with European ancestry, although detailed information about tumor type and grade in this population is limited [12–15]. There is also evidence that breast cancers in Sub-Saharan African women are more likely to lack expression of estrogen receptor (ER) [12, 13]. However, there remains a great deal of uncertainty about the distribution of ER and HER2 expression in breast cancer in Sub-Saharan Africa due to a combination of variable quality control for biomarker testing and a high level of population diversity in the region [16, 17]. Black American women have similar patterns of disease, with higher rates of high grade, ER-negative breast carcinoma and a younger age at diagnosis than their counterparts with European ancestry [5, 9, 18, 19]. All of these features are associated with more aggressive disease and worse prognosis. The reasons for ancestry-associated differences in tumor characteristics have not been well-established and may include environmental, reproductive, and genetic factors.

Recently, there has been a great deal of interest in tumor infiltrating lymphocytes (TILs) as a potential biomarker in breast cancer. Multiple studies have shown that breast cancers with prominent tumor infiltrating lymphocytes (TILs) in the adjacent stroma have an improved prognosis and are more likely to respond to specific types of chemotherapy [20–23]. Notably, the strongest associations between TIL levels and outcomes are in ER-negative tumors, which are more common in Sub-Saharan African and black American women. Additionally, ER-negative tumors have higher levels of TILs than ER-positive tumors [21, 23]. The presence of TILs is believed to reflect an active, specific anti-tumor immune response and may also identify tumors that are more likely to respond to immune-based therapies.

There has been very limited examination of tumor infiltrating lymphocytes in Sub-Saharan African populations and in populations with African ancestry in North America and Europe. Several factors indicate that the level of TILs in breast cancer could be different in women in Sub-Saharan Africa compared to North America and Europe, including differences in intrinsic properties of the tumors, population genetic differences, and differences in exposure to pathogens.

Given the high prevalence and mortality associated with breast cancer in both Sub-Saharan African women and black American women, understanding the distribution of prognostic and predictive breast biomarkers in these populations is critical. In this study, we examined the distribution of TILs and other standard biomarkers, including tumor grade and ER and HER2 expression in Tanzanian, black American, and white American populations.

## Materials and methods

We examined 90 consecutive cases of breast cancer in Tanzanian women (n = 90) at Kilimanjaro Christian Medical Centre and Bugando Medical Centre identified from each institution’s archives (timeframe 2015–2017). Among the Tanzanian cases, three failed immunohistochemical quality control tests and four had insufficient material for assessment of ER and HER2 expression and TIL analysis and were excluded (n = 83). Consecutive cases in black American (n = 120) and white American (n = 120) women were identified from the Duke Health Cancer Registry (timeframe 2016–2017). Subjects under the age of 18 and subjects who had undergone neoadjuvant chemotherapy and did not have a pre-treatment sample were excluded.

Demographic information and gross tumor characteristics were extracted from the subjects’ medical records. For each case, a representative slide was identified and assessed for tumor type, grade, mitotic count, and TIL involvement. Tumor grade was assessed according to the Elston-Ellis modification of Scarff-Bloom-Richardson grading system [24]. Mitotic counts were performed using ten high power fields, with a 0.55 mm field diameter. TIL involvement was quantified using the method recommended by the International TILs Working Group to estimate the percent stromal involvement by lymphocytes [25].

For Tanzanian cases, ER and HER2 immunohistochemical stains were performed in the clinical Image Cytometry Laboratory at Duke University Hospital, using the same methods that were used for the American cases as part of standard care. The laboratory is certified by the College of American Pathologists. ER immunohistochemistry was performed using the Dako ER pharmDx kit and Dako Link autostainer. HER2 immunohistochemistry was performed using the HercepTest kit. Cases that were equivocal for HER2 overexpression by immunohistochemistry were evaluated for HER2 amplification by fluorescent in situ hybridization. HER2 FISH was performed using the Agilent HER2IQ FISH pharmDx kit. For the American cases, ER and HER2 status was extracted from pathology reports in the subjects’ medical records.

Wilcoxon rank sum tests were used to compare TILs in groups based upon tumor grade, tumor size, and patient age. The Kruskal-Wallis test was used to compare TIL levels across all three ethnic groups. Spearman correlations were used to investigate the pairwise associations between TILs and mitotic rate. Proportions were compared using the chi-square test. P-values less than 0.05 were considered statistically significant. Statistical analyses were conducted using SAS v. 9.4 software (SAS Institute, Inc., Cary NC).

This work was approved by the Duke University institutional review board (Pro00074648), the Kilimanjaro Christian Medical University College ethics committee (research proposal 895), the Bugando Medical Centre ethics committee (CREC/185/2017) and the Tanzanian National Institute for Medical Research (NIMR/HQ/R.8a/Vol. IX/2443). Waivers of informed consent were obtained from the Duke University institutional review board, the Kilimanjaro Christian Medical University College ethics committee, and the Bugando Medical Centre ethics committee.

## Results

Breast cancer was diagnosed at a younger age in the Tanzanian group (median age 52) than in either American group (median age 62 for both American groups). Thirty-seven percent of the Tanzanian subjects were under the age of 50, compared with 23% of black American subjects and 19% of white American subjects. The Tanzanian group had more advanced disease, with larger tumors (median size 6 cm) than the black American or White American groups (2 cm and 1.8 cm, respectively) and more frequent skin ulceration (24% of Tanzanian cases, compared with none of the black American cases and 1% of the White American cases).

**Fig 1:**
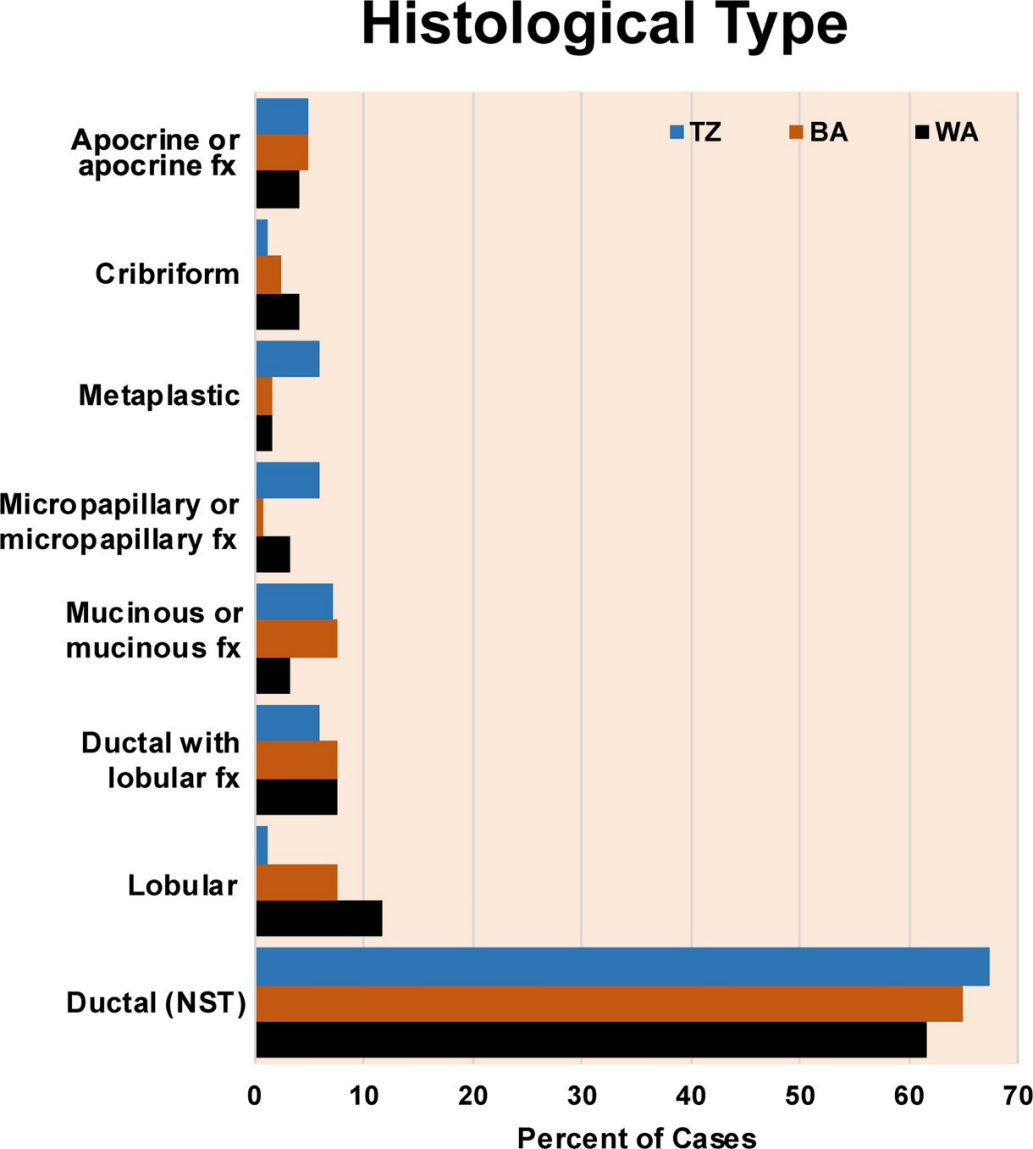
Histologic tumor type. Distribution of histologic tumor type among Tanzanian, black American, and white American women shows a predominance of ductal carcinoma of no special type among all groups and a lower frequency of lobular carcinoma in the Tanzanian group.TZ- Tanzanian, BA- black American, WA- white American, NST- no special type, fx- features.

The predominant tumor type in all three groups was ductal carcinoma of no special type (Fig 1). Lobular carcinomas were less common in the Tanzanian group than in either American group. Micropapillary and metaplastic carcinomas were more common in the Tanzanian group than in either American group, although the absolute number of these tumor types is small.

**Fig 2:**
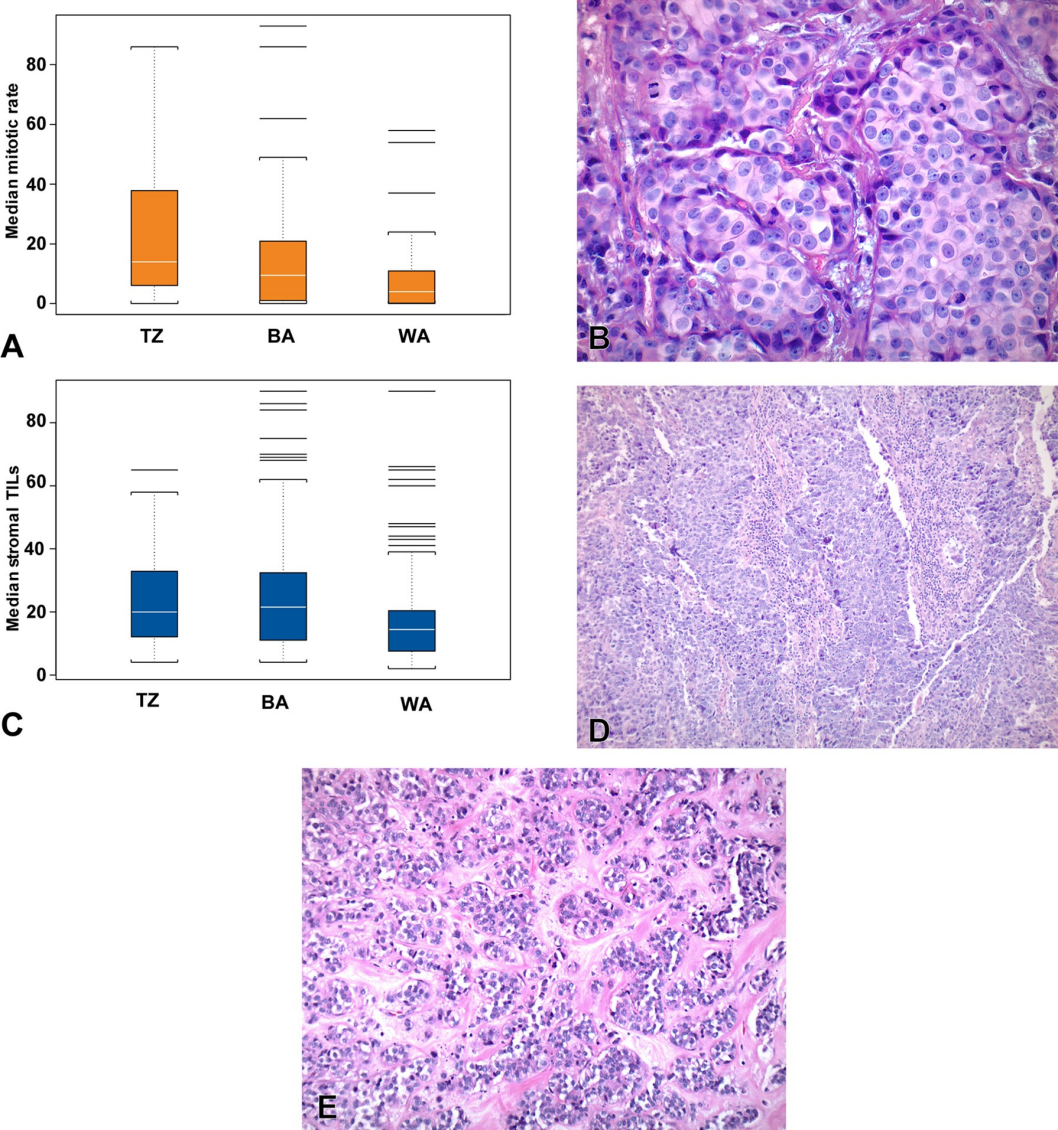
Mitotic rate and tumor infiltrating lymphocytes. A. Median mitotic rate in 10 high power fields is highest for tumors in Tanzanian women, with intermediate results in black American woman, and the lowest median mitotic rate in white American women. B. Photomicrograph of a breast cancer from a Tanzanian subject with a high mitotic rate. C. The median stromal TILs as a percentage of total stromal area in tumors is higher in Tanzanian and black American women than in white American women. D. Photomicrograph of a breast cancer from a Tanzanian subject with high stromal TILs. E. Photomicrograph of a breast cancer from a white American subject with low stromal TILs. TZ- Tanzanian, BA- black American, WA- white American.

In the Tanzanian and black American groups, there was a higher proportion of grade 3 tumors than in the white American group (Table 1, p = 0.008). When each component of the overall grade (tumor architecture, nuclear grade, and mitotic rate) was assessed separately, the most striking difference between the Tanzanian and black American cohorts and the white American cohort was in the mitotic score, which was notably higher in the Tanzanian and black American groups. The median mitotic rate in the Tanzanian group was over three-fold higher than in the white American group and the median mitotic rate in the black American group was over two-fold higher than the white American group (p<0.0001, Fig 2 and S1 Table). Even when subset by ER and HER2 status, the mitotic rate in the Tanzanian group remained notably higher (Table 2).

**Table 1:** Tumor grade and ER/HER2 status for Tanzanian, black American, and African American women. High grade tumors and ER-/HER2 tumors are more common among the Tanzanian and black American groups compared with the white American group.

	Group		
n (%)	TZ	BA	WA
**Grade 1**	9 (11%)	16 (14%)	28 (24%)
**Grade 2**	33 (40%)	47 (40%)	55 (47%)
**Grade 3**	40 (49%)	54 (46%)	35 (30%)
** **			
**ER+/HER2-**	32 (39%)	74 (62%)	93 (78%)
**HER2+**	22 (27%)	21 (18%)	15 (13)
**ER-/HER2-**	29 (35%)	25 (21%)	12 (10%)

TZ: Tanzanian, BA: black American, WA: white American

**Table 2:** Median mitotic rate in Tanzanian, black American, and African American women by biomarker type. Within all biomarker groups, the median mitotic rate is highest in Tanzanian women and lowest in white American women.

	Group		
Median (IQR)	TZ	BA	WA
**ER+/HER2-**	10.0 (2.0–16.0)	5.0 (1.0–13.0)	3.0 (0.0–9.0)
**HER2+**	18.0 (9.0–28.0)	14.0 (4.0–19.0)	9.0 (3.0–19.0)
**ER-/HER2-**	29.0 (12.0–46.0)	27.0 (14.5–41.5)	10.0 (7.0–24.0)

IQR: Interquartile Range, TZ: Tanzanian, BA: black American, WA: white American

Approximately half (51%) of the tumors in the Tanzanian cohort were positive for ER expression, a lower rate than was seen in the black American and white American cohorts (74% and 87%, respectively, p<0.001). HER2 positivity by overexpression or amplification was also significantly associated with ethnicity and was present in 27% of the Tanzanian cases, 18% of the black American cases, and 13% of the white American cases (p = 0.038). When ER and HER2 are considered together, there was a higher percentage of ER-/HER2- tumors and a lower percentage of ER+/HER2- cancers in the Tanzanian group compared with both American groups (p < 0.001, Table 1). The black American group also had a higher percentage of ER-/HER2- tumors and a lower percentage of ER+/HER2- cancers than the white American group. The Tanzanian cases were evaluated for biomarker quality control measures, including positive ER staining in benign breast tissue and rate of HER2 FISH positivity among cases with equivocal immunohistochemical staining, with appropriate results (S1 Fig).

The median stromal TIL level was higher in the Tanzanian and black American groups than in the white American group (p = 0.0001, Fig 2). However, when subset by ER and HER2 status, the differences were not statistically significant (p = 0.78). Within all three groups, TIL levels were weakly to moderately positively correlated with mitotic rate (r = 0.25 to 0.50). Among Tanzanian and black American subjects, TIL levels were significantly higher in ER-negative tumors (both p = 0.02).

When evaluation was limited to subjects under age 50, there are similar trends to those in the group as a whole. Specifically, Tanzanian and black American subjects still had higher grade tumors, higher mitotic rates, fewer ER-positive tumors, and higher levels of TILs. The differences were particularly stark in terms of ER and HER2 status in the subjects under 50, with less than one third as many ER+/HER2 tumors and more than twice as many ER-/HER2- tumors in the Tanzanian group compared to the white American group (Table 3).

**Table 3:** Tumor characteristics for subjects under 50 years old among Tanzanian, black American, and African American women. In women under 50, there are similar trends to the group as a whole, with higher grade tumors, fewer ER+/HER2- tumors, higher mitotic rates, and higher levels of TILs in Tanzanian and African American women compared to white American women.

	Group		
n (%)	TZ	BA	WA
**Grade 1**	2 (6%)	3 (12%)	3 (13%)
**Grade 2**	11 (34%)	9 (35%)	13 (57%)
**Grade 3**	19 (59%)	14 (54%)	7 (30%)
** **			
**ER+/HER2-**	6 (19%)	14 (52%)	17 (74%)
**HER2+**	8 (25%)	7 (26%)	2 (9%)
**ER-/HER2-**	18 (56%)	6 (22%)	5 (17%)
Median (IQR)			
**Mitotic rate**	14.5 (9.0–29.0)	15.0 (4.0–29.0)	4.0 (1.0–13.0)
**TILs**	21.5 (12.5–30.5)	23.0 (15.0–31.0)	16.0 (8.0–20.0)

IQR: Interquartile Range, TZ: Tanzanian, BA: black American, WA: white American

## Discussion

This study demonstrates that Tanzanian and black American women are more likely to have tumors with aggressive features such as high tumor grade, high mitotic rate, and ER negativity than white American women, but that levels of TILs, typically associated with a better prognosis, are also higher in the Tanzanian and black American cohorts.

The high grade of the carcinoma in Tanzanian and black American women was largely driven by high mitotic rates. Previous studies have shown that carcinomas with a high mitotic index are associated with shorter survival and an increased risk of distant recurrence [26]. Conversely, carcinomas with more frequent mitoses tend to be more responsive to chemotherapy. Vincent-Salomon, et al. showed a strong correlation between breast carcinoma mitotic rate and response to neoadjuvant chemotherapy [27]. Among subjects with a mitotic index of more than 17 mitoses in 10 high power fields, half of all subjects had a pathological complete response. In contrast, the pathological complete response rate was only 7% among subjects with a lower mitotic index. Accounting for differences in field diameter, 56% of the Tanzanian cases in this study have a mitotic rate that would place them in the high response rate group, compared with 42% of black American cases and 21% of White American cases. Further evaluation into the role of the mitotic index as a predictive marker for chemotherapy response in Sub-Saharan African and black American populations is warranted. In addition, investigation into the molecular basis for high mitotic rates may yield insight into breast cancer biology in Tanzanian and black American populations.

Previous studies of hormone receptor expression in Sub-Saharan Africa have reported widely varying results, with estrogen receptor expression ranging from 14% to 76% [16]. A major limitation to the interpretation of prior studies has been uncertainty about quality control for the immunohistochemical stains in terms of both pre-analytic and analytic variables. In order to assess the accuracy of the immunohistochemical stains performed on the samples from the Tanzanian cohort, all tests were performed in a College of American Pathologists-certified laboratory, with external positive controls and evaluated for ER staining in benign breast epithelium in the study tissue as an internal positive control (S1 Fig). We found a small number of cases with negative staining in benign epithelium (3 of 90 cases). These cases also had morphologic features of poor fixation and were excluded from further analysis. For HER2 immunohistochemical staining, there is no internal positive control. However, one way to assess test accuracy is the rate of FISH positivity among cases with equivocal results. A total of 8 Tanzanian cases had equivocal results by immunohistochemisty, of which 2 were positive for amplification by FISH. These results fall within the reported range of 14–33% HER2-amplified cases among breast cancers with equivocal results by HER2 immunohistochemistry [28, 29]. Based on these quality control measures, we feel that the data on immunohistochemical staining in this study is reliable. In addition, the presence of higher grade tumors with higher mitotic rates in the Tanzanian group is consistent with the presence of lower rates of ER expression and higher rates of HER2 positivity in the group.

The frequency of ER-/HER2- tumors was higher in the Tanzanian group than in the black American group, which in turn had a higher rate than the white American group. The reasons for these ancestry-associated differences are uncertain, although there is some evidence that environmental and genetic factors may play a role [30, 31]. For example, multiparity and younger age at first full-term pregnancy are associated with a lower risk of ER-positive breast cancer, but an increased risk of ER-negative disease [31]. Recent studies have also identified several novel genetic variants associated with ER-negative breast cancer in black Americans that were not identified in studies of European ancestry populations [32, 33]. In addition, there is increasing evidence that psychosocial stressors, including those associated with poverty and racial discrimination, can cause damage at the cellular level and may contribute to biological differences in cancer [34, 35]. Another major difference between the Tanzanian cohort and the American cohorts is that all of the Tanzanian cohort had clinically apparent lesions, whereas many of the American cases were detected by screening mammography. As ER-/HER2- tumors tend to grow quickly, they are more likely to be detected clinically [36].

The frequency of ER-/HER2- tumors in the present study (35%) is similar to prior studies of breast cancer in Tanzania and Kenya [12, 13, 15]. Recent studies have suggested that the frequency of ER-/HER2- tumors in sub-Saharan Africa may have significant regional variation, with a relatively high proportion ER-/HER2- tumors in West Africa (Ghana, 53%) and lower proportions in East Africa (Ethiopia, 15%) and South Africa (20% among black women) [17, 37]. It is worth noting that there is a great deal of genetic diversity in East Africa and there is evidence that some Kenyan and Tanzanian populations are genetically quite distinct from Ethiopian populations and may be more similar to West African populations [38]. The degree of geographic variation in ER-status demonstrates the need for an expanded examination of breast cancer across the diverse populations of Sub-Saharan Africa.

In this study, the frequency of ER-/HER2- tumors in the black American cohort (21%) is lower than in some prior studies, which found that 30% to 39% of breast cancers in black American women were ER-/HER2- [17, 18]. One reason for this discrepancy may be the timeframe for the case ascertainment in the prior studies, which is 10 to 24 years earlier than the cases in this study. During this time, screening imaging has become more sensitive and more frequently used, resulting in the identification of more small, relatively indolent tumors, which are frequently ER-positive. In addition, biomarker testing techniques and interpretation guidelines have changed, which also affects the rate of tumors testing positive for ER. The frequency of ER-/HER2- tumors in the black American cohort is also lower than the reported rates of ER-/HER2- disease in West Africans [17]. This is notable because most black Americans have West African ancestry.

The frequency of HER2-positivity was quite high in the Tanzanian cohort, compared with the black American and particularly the white American cohorts. Similar to ER-/HER2- breast cancers, HER2+ cancers tend to grow rapidly. Therefore, some of this difference may be explained by the predominance of clinically apparent tumors in the Tanzanian group. A similarly high rate of HER2 positivity was previously reported in a study of breast cancer in Ethiopia [17].

Breast cancers in Tanzanian and black American subjects had a higher rate of TIL involvement than breast cancers in white American subjects. This difference appears to be primarily due to, the increased frequency of ER-negative tumors in the Tanzanian and black American populations, which are known to have higher levels of TILs than ER-positive tumors. When we analyzed only ER- tumors, there was no statistically significant difference between the groups. It is possible that other factors, including environmental influences and differences in exposure to infectious agents could also affect the anti-tumor immune response. Evaluation of larger numbers of ER- tumors in future studies will be helpful to determine if there are more subtle difference between the groups.

In studies performed on primarily European ancestry populations, increasing levels of TILs were associated with better response to chemotherapy and better prognosis [20–23]. However, some studies have suggested that black American women have a poorer response to neoadjuvant chemotherapy than White American women [39]. Given the high levels of TILs in breast cancers in both Tanzanian and black American women, but relatively poor outcomes in both groups, additional studies into the nature of the immune response and the association between TILs and outcomes in these populations is necessary.

Having a better understanding of the distribution of biomarker profiles in Tanzanian women provides key information for determining health care priorities for the population. While the rate of ER expression in breast carcinomas from Tanzanian women was low compared to either American population, approximately half of all tumors in the Tanzanian group were ER-positive, indicating that a significant proportion of Tanzanian breast cancer patients would benefit from hormonal therapy. In addition, over one quarter of breast cancers in the Tanzanian women in this study were HER2-positive and would be expected to have better outcomes with HER2-directed therapy. Furthermore, based on high mitotic rates and high levels of TILs seen in the Tanzanian cohort, many Tanzanian breast cancer patients would be expected to derive advantages from cytotoxic chemotherapy in general and neo-adjuvant chemotherapy in particular. These findings highlight the urgent need for access to ER and HER2 testing and hormonal therapy, HER2-directed therapy, and cytotoxic chemotherapy in the region to allow for the most efficient and highest quality care.

## Supporting information

S1 Fig Photomicrograph of an ER-negative breast cancer from a Tanzanian subject, with positive staining in benign ducts as an internal positive control. (TIF)

S1 Table Mitotic rate and TILs for all subjects. IQR: Interquartile range, TZ: Tanzanian, BA: black American, WA: white American. (DOCX)
