# Supplementary figures and images for: Breast cancer in Tanzanian, black American, and white American women: An assessment of prognostic and predictive features, including tumor infiltrating lymphocytes
Source: PLoS One. 2019 Nov 8;14(11):e0224760. doi: 10.1371/journal.pone.0224760 (PMC6839867; doi:10.1371/journal.pone.0224760)

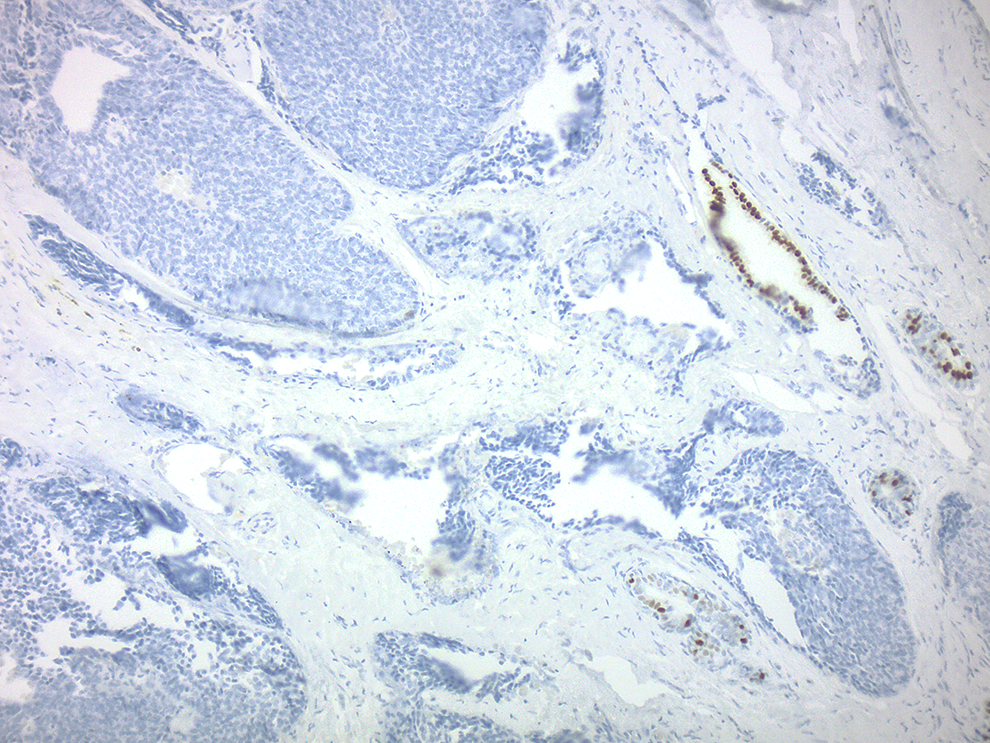

Supplement: S1 Fig — (TIF) [file pone.0224760.s001.tif]
