# Supplementary material for: Breast cancer in Tanzanian, black American, and white American women: An assessment of prognostic and predictive features, including tumor infiltrating lymphocytes
Source: PLoS One. 2019 Nov 8;14(11):e0224760. doi: 10.1371/journal.pone.0224760 (PMC6839867; doi:10.1371/journal.pone.0224760)
Supplement: S1 Table — (DOCX) [file pone.0224760.s002.docx]

**Supplemental Table 1.** Mitotic rate and TILs for all subjects.

|  | **Group** | | |
| --- | --- | --- | --- |
| Median (IQR) | **TZ** | **BA** | **WA** |
| Mitotic rate | 14.0 (6.0-38.0) | 9.5 (1.0-21.0) | 4.0 (0.0-11.0) |
| TILs | 20.0 (12.0-33.0) | 21.5 (11.0-32.5) | 14.5 (7.5-20.5) |

IQR: Interquartile range, TZ: Tanzanian, BA: black American, WA: white American
